# LXRβ controls glioblastoma cell growth, lipid balance, and immune modulation independently of ABCA1

**DOI:** 10.1038/s41598-019-51865-8

**Published:** 2019-10-29

**Authors:** Deven Patel, Fahim Ahmad, Diane M. Kambach, Qian Sun, Alan S. Halim, Tamalee Kramp, Kevin A. Camphausen, Jayne M. Stommel

**Affiliations:** Radiation Oncology Branch, National Cancer Institute, National Institutes of Health, Bethesda, MD 20892 USA

**Keywords:** CNS cancer, Lipid signalling

## Abstract

Cholesterol is a critical component of membranes and a precursor for hormones and other signaling molecules. Previously, we showed that unlike astrocytes, glioblastoma cells do not downregulate cholesterol synthesis when plated at high density. In this report, we show that high cell density induces ABCA1 expression in glioblastoma cells, enabling them to get rid of excess cholesterol generated by an activated cholesterol biosynthesis pathway. Because oxysterols are agonists for Liver X Receptors (LXRs), we investigated whether increased cholesterol activates LXRs to maintain cholesterol homeostasis in highly-dense glioblastoma cells. We observed that dense cells had increased oxysterols, which activated LXRβ to upregulate ABCA1. Cells with CRISPR-mediated knockdown of LXRβ, but not ABCA1, had decreased cell cycle progression and cell survival, and decreased feedback repression of the mevalonate pathway in densely-plated glioma cells. LXRβ gene expression poorly correlates with ABCA1 in glioblastoma patients, and expression of each gene correlates with poor patient prognosis in different prognostic subtypes. Finally, gene expression and lipidomics analyses cells revealed that LXRβ regulates the expression of immune response gene sets and lipids known to be involved in immune modulation. Thus, therapeutic targeting of LXRβ in glioblastoma might be effective through diverse mechanisms.

## Introduction

Glioblastoma, or GBM, is the most common malignant primary brain tumor and among the most lethal of all cancers^1,2^. GBM has a median survival of 15 months with current standard of care therapy, which includes radiation, surgery, and temozolomide^3^. Because of the invasive nature of GBM, the entire tumor cannot be removed surgically. The Cancer Genomic Atlas (TCGA) project has revealed interesting insights regarding genes and pathways that are altered in GBM, such as the RTK/PI3K/MAPK (90%), p53 (86%), Rb pathways (79%)^4^. Despite the prevalence of “druggable” target alterations in GBM, small molecule therapies have yet to make any inroads into this disease. There are multiple possible reasons for the failure of these therapies such as pathway redundancy^5^, genomic heterogeneity between tumor cells^6,7^, and preventive effects of the blood brain barrier. These complications call for better understanding of the biology of these tumors to find novel therapies.

Contact inhibition is a key process in the transformation of normal cells to malignancy^8^. Normal cells use contact inhibition to maintain tissue homeostasis in the body. In contrast, cancer cells are unable to arrest proliferation at confluence^8^. In fact, loss of contact inhibition that leads to uncontrolled proliferation is used as an *in vivo* prognostic factor in human cancer^9^. Cancer cells grown at high density are resistant to a diverse array of cytotoxic cancer therapeutics such as anthracyclines, antibiotics, vinca alkaloids, taxanes, nitrosureas and bleomycin^10–12^. In normal cells, cell-cell contact negatively affects growth factor-mediated intracellular signaling pathways, such as ERK and Akt, to suppress cell cycle progression^13^. Besides its role in promoting cell division, Akt activity also leads to transcription of the enzymes involved in cholesterol and fatty acid biosynthesis via the sterol regulatory element-binding protein (SREBP) transcription factors^14^, both critical components of membranes and signaling pathways needed to maintain growth and proliferation. The regulation of cholesterol homeostasis by cell density is dysregulated in glioblastoma: at high cell density, normal astrocytes turn off cholesterol synthesis and reduce the levels of cholesterol while glioblastoma cells ignore density-dependent regulation and maintain cholesterol synthesis^15^.

Cholesterol is an important nutrient for normal cell function and viability. It plays a critical role in the plasma membrane and lipid rafts and act as a precursor for steroid hormones, bile acids, and Vitamin D. In the brain, cholesterol is synthesized locally because exogeneous cholesterol cannot cross the blood brain barrier. In the central nervous system, cholesterol synthesis and clearance are regulated to create a tightly coupled homeostatic system that allows a modest amount of cholesterol turnover while keeping the overall levels consistent^16^. Cholesterol metabolism in mammals is regulated through the coordinated actions of SREBP and Liver X Receptor (LXR) transcription factors^17–19^. SREBPs induce the genes associated with cholesterol biosynthesis and enhance the uptake of extracellular cholesterol by induction of Low-Density Lipoprotein Receptors (LDLRs)^20^. LXRs responds to excess cholesterol in the cells by activating the transcription of the cholesterol efflux transporters, *ABCA1* and *ABCG1*^21^. LXRs also down-regulate the uptake of cholesterol by inducing the synthesis of IDOL (Inducible Degrader of LDLR), which is an E3 ubiquitin ligase that mediates the degradation of LDLR^22^. An LXRα binding site in the proximal promoter region of the rat 7A-hydroxylase CYP7A also promotes the removal of cholesterol by increasing its conversion to bile acids^23^.

Under physiologic conditions, oxysterols such as 22(R)-hydroxycholesterol, 24(S),25-epoxycholesterol, and 24(S)-hydroxycholesterol, strongly induce LXRα and LXRβ transcriptional targets but not other sterols (lanosterol, desmosterol, steroid hormone precursors, testosterone, progesterone, or bile acids)^24^. 24-OHC, the most abundant oxysterol in the brain^25^, is generated by hydroxylation of cholesterol in neurons by the cytochrome P450 enzyme cholesterol 24-hydroxylase (CYP46A1) and gets cleared in an ABCA1-dependent manner^26,27^. It subsequently crosses the blood-brain barrier by passive diffusion across membranes^28^. In the brain, cholesterol homeostasis is maintained by delicately balancing 24-OHC levels. Physiological concentrations of 24-OHC induce LXR signaling, generating a neuroprotective response^29,30^. At higher concentrations, 24-OHC inhibits LXR transcriptional activity^29^ and promotes “necroptosis-like” death pathway in neurons^31^.

Several synthetic LXR agonists, including T0901317, GW2965, and LXR-623, have been shown to increase the expression of LXR target genes and alter circulating lipid levels in rodent models^32–34^. LXR-623 was shown to target GBM by lowering cellular cholesterol content through the upregulation of the cholesterol efflux transporter, ABCA1, thus disrupting cholesterol homeostasis^35^. Because we previously found that *de novo* cholesterol synthesis is upregulated in patient-derived glioma tumor neurospheres^15^, we explored below the hypothesis that inhibiting LXR-mediated cholesterol homeostasis might increase cholesterol levels to lethal levels in glioma cells. We found that LXRβ enables glioma cells to proliferate and survive at high cell densities when cholesterol is high and represses feedback through the mevalonate pathway. Interestingly, this did not appear to work solely through its major downstream effector ABCA1, as CRISPR-mediated knockdown of this gene did not recapitulate the cellular phenotypes observed with knockdown of LXRβ. In the glioma tumor initiating cells, LXRβ activated transcription of *ABCA1*, but also immune modulation pathways and the production of glycerophospholipids. These studies provide a further rationale for exploring LXR signaling as a novel therapeutic intervention in GBM.

## Results

### Glioma cells upregulate ABCA1 high cell density

**Figure 1:**
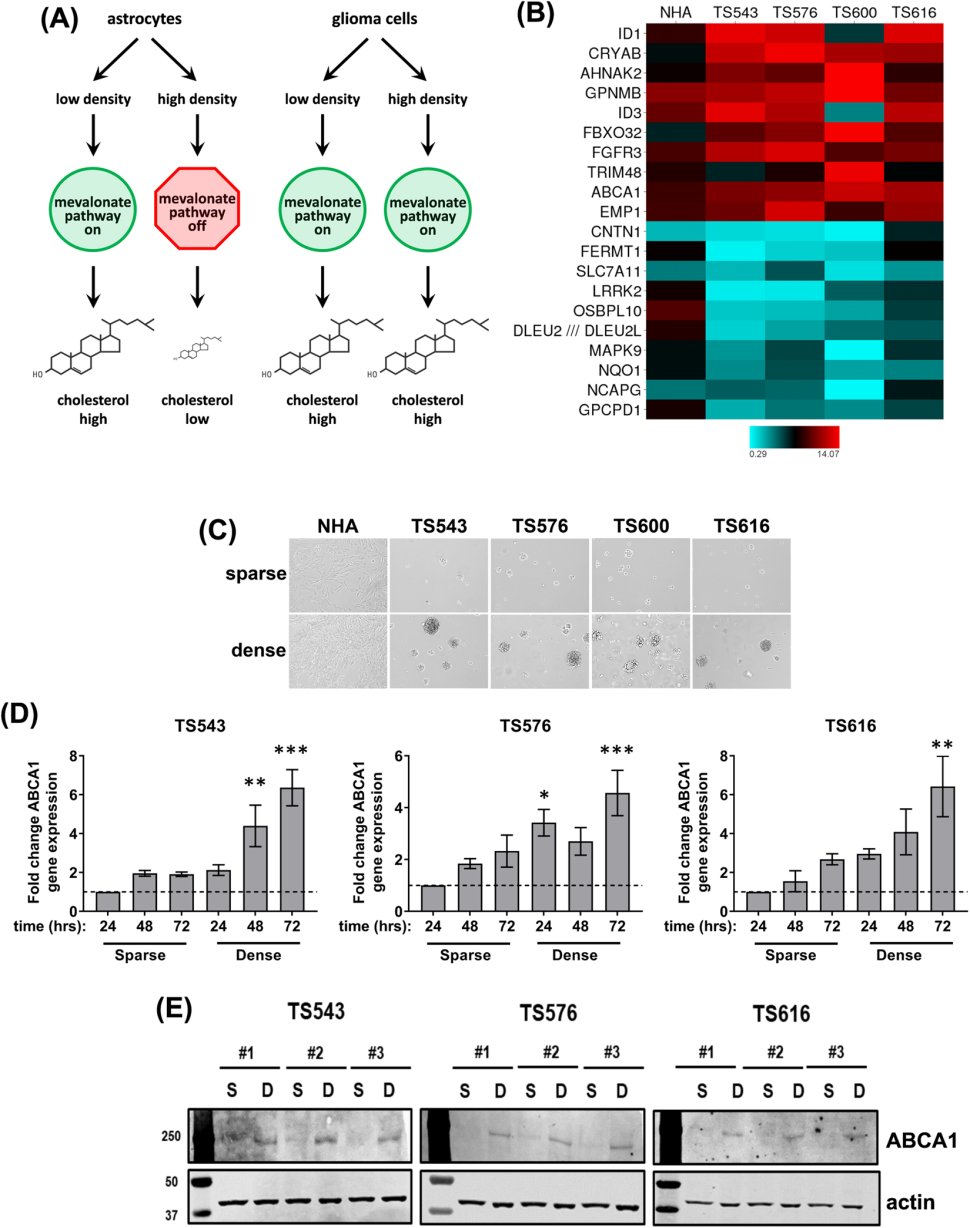
Glioma cells plated at high density induce ABCA1 to maintain cholesterol homeostasis. (**A**) Density-dependent regulation of cholesterol synthesis in astrocytes vs. glioma cells. Based on data from Kambach *et al*.^15^. (**B**) Heat map of density-dependent gene expression from microarrays of normal human astrocytes (NHA) and glioma tumor initiating cells. Each value is the ratio of dense/sparse gene expression. The top and bottom 10 differentially expressed gene averaged across all four glioma lines is shown. (**C**) Phase contrast image of glioma tumor initiating cells were plated at low (sparse) and high (dense) densities. (**D**) Relative gene expression analysis for *ABCA1* in TS543, TS576, and TS616 glioma cells. Gene expression values were derived from quantitative real time PCR normalized to *GAPDH* and expressed relative to the 24 hour time point for sparse cells. Error bars indicates SEM for at least 3 replicates. *p < 0.05, **p < 0.005, ***p < 0.0005 versus 24 hour sparse by one-way ANOVA with Dunnett’s multiple comparisons test. (**E**) Western blot analysis of ABCA1 and β-actin in TS543, TS576 and TS616 glioma cells comparing sparse vs. dense conditions for three biological replicates (#1–3).

A study published earlier from our lab showed that glioma cells have dysregulated cholesterol synthesis when compared to normal astrocytes^15^. In our previous work, we found that glioma cells constitutively express the mevalonate pathway and keep cholesterol levels high at high cell densities. In contrast, normal human astrocytes (NHAs) significantly downregulate genes in the mevalonate pathway and decrease total cellular cholesterol when grown to high cell density and contact-inhibited (see ref.^15^ and Fig. 1A). We were interested in exploring the impact of dysregulated cholesterol synthesis on glioma cell biology. We first examined gene expression profiles from patient-derived primary glioma tumor neurosphere cells (TS543, TS576, TS600, and TS616) and NHAs plated at low and high density. The cholesterol efflux transporter, *ABCA1*, was among the genes with the highest induction in glioma cells plated at high density, with an average rank = #9, (TS543: 1.7x induction, p = 0.001, rank = #96; TS576: 2.2x induction, p = 0.00001, rank = #22; TS600: 4.6x induction, p = 0.00005, rank = #10; TS616: 2.8x induction, p = 0.00005, rank = #8; Fig. 1B,C). We confirmed this result with quantitative real time PCR, plating glioma cells at low (sparse) or high (dense) density and measuring *ABCA1* RNA expression levels 24, 48, or 72 hrs after plating (Fig. 1D). *ABCA1* RNA levels were higher in cells plated at high density, and as cells became denser through proliferation in culture. The RNA levels of another ATP-binding cassette cholesterol efflux transporter, *ABCG1*, were not above background when measured on the microarrays or by real time quantitative PCR. We also measured the protein levels of ABCA1 on a western blot and saw that its levels significantly increased at higher density across multiple cell lines (Fig. 1E).

The NHAs also had a slight and less significant induction of *ABCA1* at high cell density on the microarrays (NHA: 1.2x induction, p = 0.08, rank = #2964; Fig. 1B,C) and this was confirmed to be reproducible by quantitative real time PCR and immunoblotting (Figures S1A,B). Together, these experiments suggest that while the cholesterol efflux transporter ABCA1 is upregulated in both the glioma cells and the normal astrocytes at high cell density, only the glioma cells keep cholesterol levels high through compensatory *de novo* cholesterol biosynthesis via the mevalonate pathway.

### LXRβ is activated to upregulate ABCA1 at high glioma cell density

**Figure 2:**
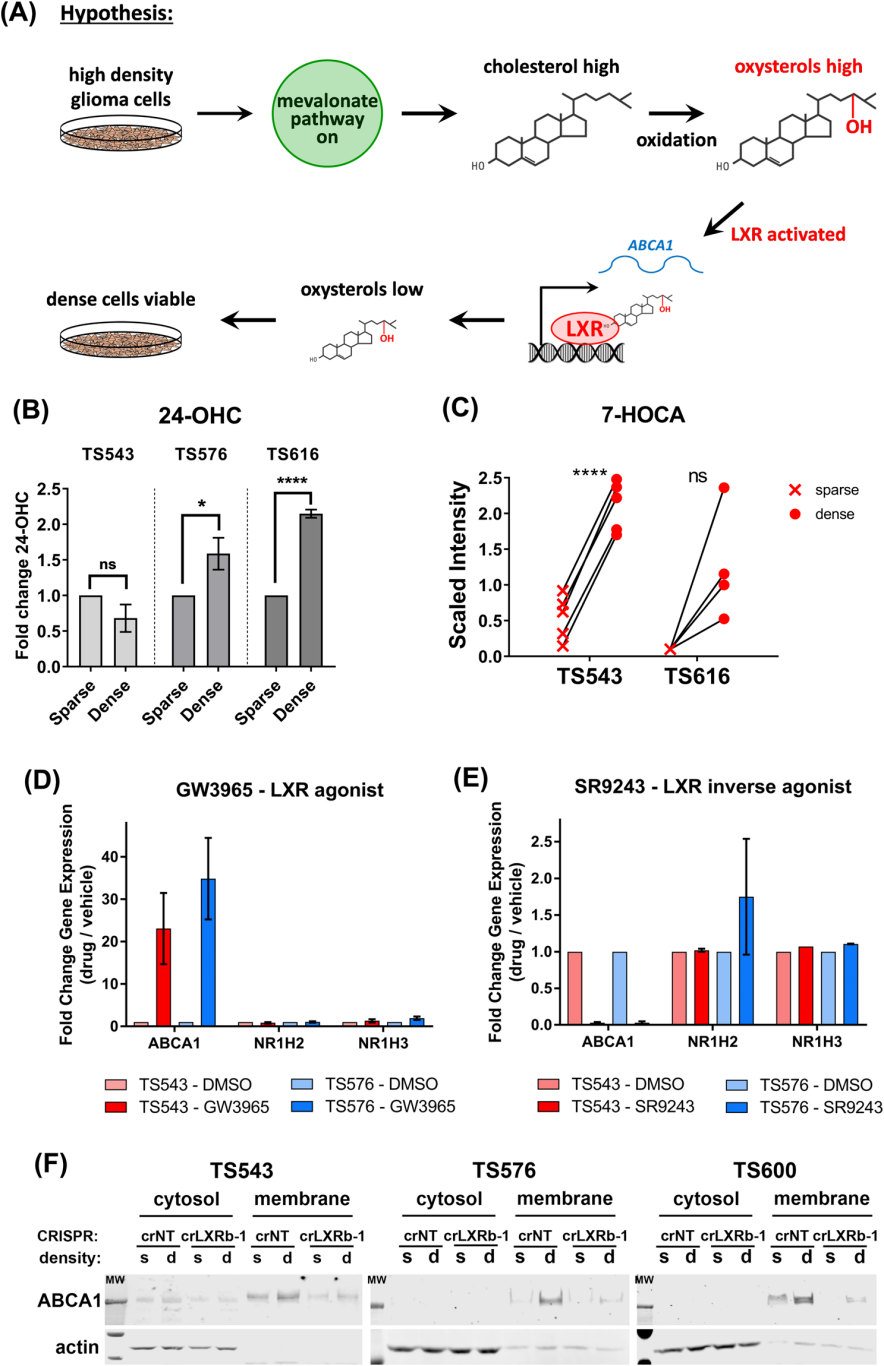
Higher density leads to activation of LXRβ in glioma TS cells. (**A**) Hypothetical mechanism by which LXRβ maintains the viability of densely plated glioma cells. High cell density leads to increased intracellular cholesterol levels through activation of the mevalonate pathway. Oxysterols, activating ligands for LXRβ, are generated through intracellular oxidation of cholesterol. Activated LXRβ turns on the expression of genes that lower intracellular cholesterol, including the cholesterol efflux transporter, *ABCA1*. Cells remain viable due to the decreased levels of cholesterol and oxysterols. (**B**) Fold change in 24-Hydroxycholesterol (24-OHC) levels in TS543, TS576 and TS616 glioma cells. Levels were normalized to sparse for each cell line. Error bars indicates SEM for at least 3 experiments. *p < 0.05; ****p < 0.0001 for One Way ANOVA of sparse vs. dense. (**C**) Fold change in 7-HOCA levels in TS543 and TS616 glioma cells. Each x or closed circle represents an independent measurement. **** =  < 0.0001 for paired t-test of sparse vs. dense (**D**) Quantitative real time PCR of *ABCA1*, *NR1H2* (LXRβ), and *NR1H3* (LXRα) in TS543 and TS576 cells treated for 24 hrs with 5 μM GW3965 or DMSO control. Data are the average of 3 biological replicates and are normalized to GAPDH and untreated for each gene and cell line. Bars = SEM. (**E**) Quantitative real time PCR of *ABCA1*, *NR1H2*, and *NR1H3* in TS543 and TS576 cells treated for 24 hrs with 5 μM SR9243 or DMSO control. Data are the average of 2 biological replicates and are normalized to *GAPDH* and untreated for each gene and cell line. Bars = SEM. (**F**) Western blot analysis of ABCA1 in TS543, TS576 and TS600 LXRBβ CRISPR knockout cells comparing sparse vs dense conditions in fractions of cytosolic vs membrane components. Data shown are representative of at least 3 biological replicates.

Cholesterol in cells is oxidized to oxysterols, which can be cytotoxic at high levels^36–38^. Oxysterols activate the Liver X Receptors, LXRα and LXRβ, to turn on the expression of genes such as *ABCA1* that lower cellular cholesterol levels^24,39^. We therefore hypothesized that LXR might maintain the viability of glioma cells with constitutively activated cholesterol biosynthesis^15^ by reducing cytotoxic cholesterol levels (Fig. 2A). We first measured the levels of oxysterols and oxysterol metabolites in glioma cells grown at high and low cell density. We observed that at high plating density, two of three glioma lines had significantly higher levels of 24-OHC, the predominant oxysterol present in the brain^25^, (Fig. 2B). TS543 cells, which did not increase 24-OHC at high density, instead increased the levels of 7-HOCA (7alpha-Hydroxy-3-oxo-4-cholestenoate), a major brain metabolite of 27-OHC^40^ that we were unable to detect in these cells, suggesting that LXR might be activated by 27-OHC in this line (Fig. 2C).

We next determined whether LXR turns on the expression of ABCA1 in our glioma tumor neurosphere cells. We saw that a synthetic LXR agonist, GW3965^33^, increased *ABCA1* RNA levels but did not change either *NR1H2* or *NR1H3* (the genes encoding LXRβ and LXRα) in either of two different glioma lines, TS543 and TS576 (Fig. 2D). Conversely, a synthetic LXR inverse agonist, SR9243^34^, decreased *ABCA1* RNA levels without affecting either *NR1H2* or *NR1H3* (Fig. 2E).

To determine which LXR paralog was likely to be activating ABCA1 in our glioma cells, we used the UCSC Xena browser to look at expression levels of *NR1H2* and *NR1H3* in normal tissues in the Genotype-Tissue Expression (GTEx) data set. Most tissues, including brain, expressed *NR1H2* (protein = LXRβ) at higher levels than *NR1H3* (Figure S2A). We therefore generated glioma neurosphere lines with CRISPR-mediated knockdown of *NR1H2* (Figure S2B,C). Glioma cells with CRISPR-modified *NR1H2* (crLXRβ-1) expressed less ABCA1 in membrane fractions of cells at high cell density than cells modified by a non-targeting CRISPR control (crNT) (Fig. 2F). Together, the above data suggest that high cell density leads to activation of LXRβ which in turn increases expression of the cholesterol efflux pump, ABCA1.

### Glioma cells are dependent on LXRβ signaling to maintain cholesterol homeostasis, cell viability, and cell proliferation

**Figure 3:**
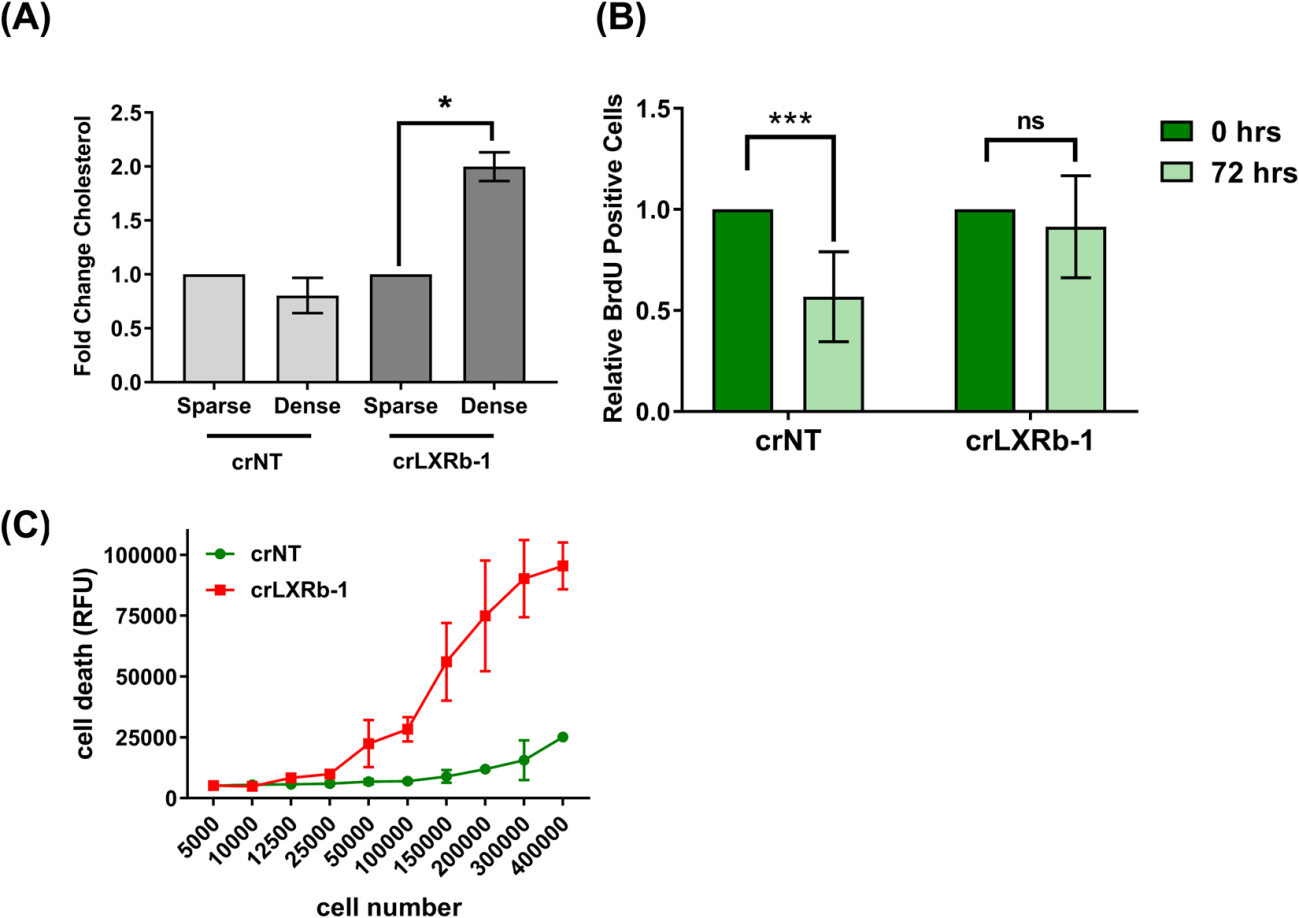
Glioma cells rely on LXRβ signaling to maintain cholesterol, cell viability and cell proliferation. (**A**) Fold change in cholesterol levels in glioma TS600 LXRβ CRISPR knockout cells (crLXRb-1) and non-targeting control (crNT). For each knockout condition, levels were normalized to sparse. Error bars indicate SEM for at least 3 experiments. *p < 0.05 for paired t-test of sparse vs. dense. (**B**) BrdU cell proliferation assay in TS600 crNT and crLXRb-1 cells. BrdU quantitation is normalized to sparse for each knockout condition. Error bars indicate SEM for at least 3 replicates. ***p = 0.0008 for two-tailed unpaired t-test. (**C**) Cell death in TS600 crNT and crLXRb-1 cells plated at increasing cell numbers, as measured with CellTox Green. Error bars indicate SD for at least 4 replicates. Data shown are representative of at least 3 biological replicates.

We speculated that since cells at higher density have increased ABCA1 transporters to remove excess cholesterol, cells without LXRβ should accumulate cholesterol, possibly negatively effecting cell viability (see Fig. 2A). We used an Amplex Red assay to measure cholesterol levels and found that in the absence of LXRβ, cells at higher plating density have increased cholesterol (Fig. 3A), presumably due to decreased ABCA1-mediated cholesterol transport under conditions in which ABCA1 levels are normally increased. We investigated the consequences of cholesterol accumulation on cell cycling and viability. We performed a BrdU dilution assay in TS600 crLXRβ tumor neurosphere cells: we pulsed cells with BrdU for 4 hours, then we measured the extent to which the incorporated BrdU was diluted by cell division 72 hours later. We observed that while the crNT cells had less incorporated BrdU 72 hrs after pulsing, in the absence of LXRβ the BrdU was not diluted (Fig. 3B), suggesting that LXRβ is necessary for division of glioma cells plated at high density. We further performed a CellTox Green assay to check the effect of cholesterol accumulation on cell viability. As shown in Fig. 3C, as the cells were grown in increasingly dense conditions, the absence of LXRβ led to increased cell death compared to cells expressing a non-targeting control. Therefore, by modulating cholesterol levels, LXRβ enables continued proliferation and viability under conditions of high density.

### Cholesterol homeostasis is mediated by LXRβ signaling independent of ABCA1

**Figure 4:**
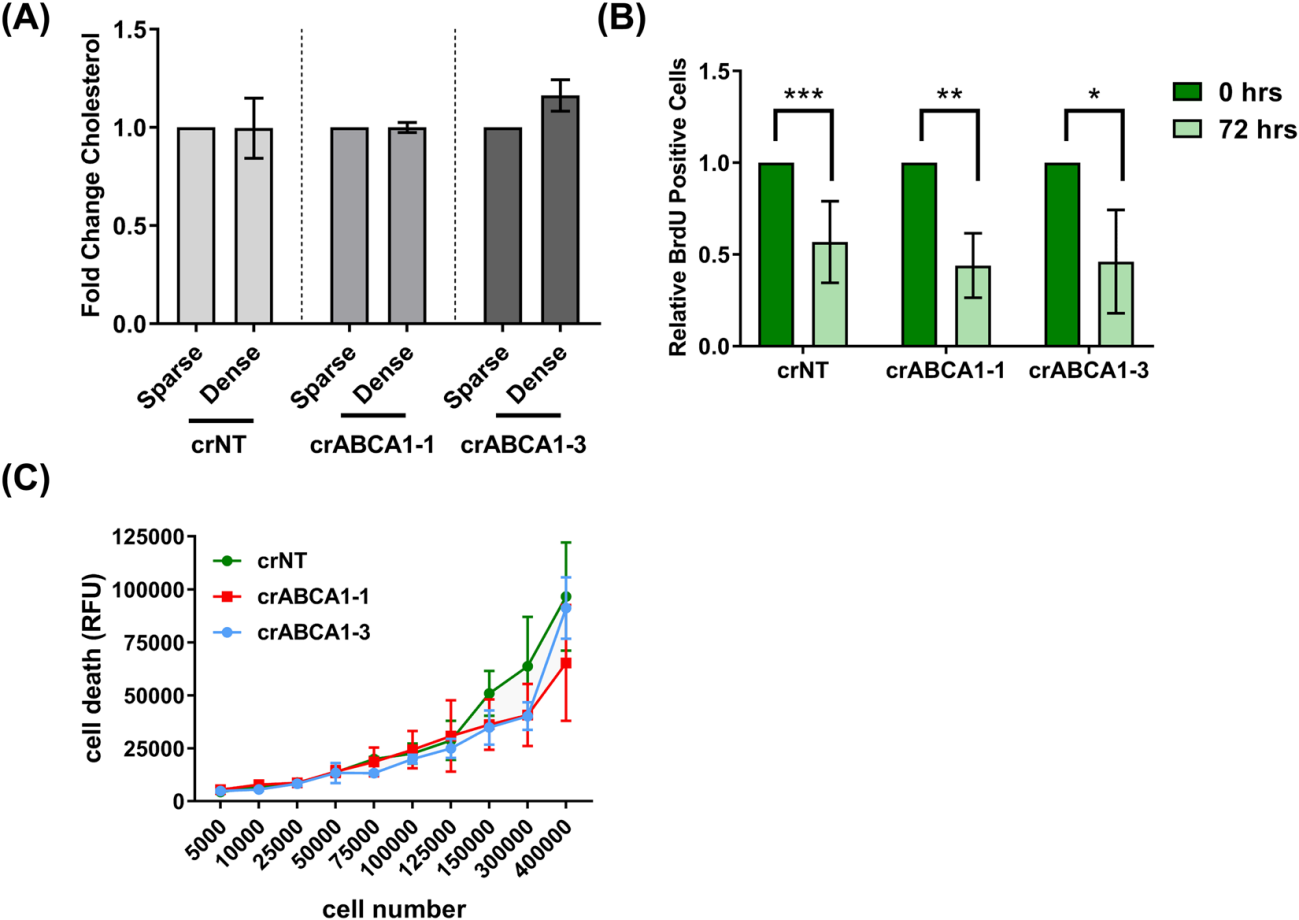
ABCA1 expression is not essential for overall cholesterol levels, cell proliferation, or cell viability. (**A**) Fold change in cholesterol levels in glioma TS600 ABCA1 CRISPR knockout cells (crABCA1-1 and crABCA1-3) and non-targeting control (crNT). For each knockout condition, levels were normalized to sparse. Error bars indicate SEM for at least 3 experiments. (**B**) BrdU cell proliferation assay in TS600 crNT and crABCA1 cells. BrdU quantitation is normalized to sparse for each knockout condition. Error bars indicate SEM for at least 3 replicates. ***p = 0.0008, **p = 0.005, *p = 0.03 for two-tailed unpaired t-test. (**C**) Cell death in TS600 crNT and crABCA1 cells plated at increasing cell numbers, as measured with CellTox Green. Error bars indicate SD for at least 4 replicates. Data shown are representative of at least 3 biological replicates.

We next investigated whether the functional role of LXRβ signaling is mediated exclusively by the cholesterol efflux transporter, ABCA1, or by other downstream effectors in the LXRβ pathway. To test this hypothesis, we developed ABCA1 knock-out cells using CRISPR-Cas9 technology (Fig S2D). As shown in Figure S2E, ABCA1 protein levels were reduced at high cell density in the ABCA1 CRISPR cells relative to a non-targeting CRISPR control (crNT). We next checked the levels of cholesterol in the crABCA1 cells at low and high cell density. Unlike the crLXRβ cells in Fig. 3A, the crABCA1 cells did not increase cholesterol levels at high density despite their reduced ABCA1 (Fig. 4A). We next investigated the impact on cell cycle progression of the crABCA1 glioma cells. In contrast with crLXRβ shown in Fig. 3B, when we performed a BrdU dilution assay with the crABCA1 cells, we observed that BrdU continued to be diluted throughout the course of the experiment, indicating that the crABCA1 cells are progressing through the cell cycle similarly to the crNT cells (Fig. 4B). Also in contrast with crLXRβ, we observed no significant difference in cell death in between crNT and crABCA1 cells plated at increasing density (Fig. 4C). Overall, these observations that the effects of CRISPR-mediated knockdown of LXRβ are not fully recapitulated by ABCA1 are consistent with two explanations: (1) that ABCA1 is a necessary but not a sufficient mediator of LXRβ signaling and/or (2) that cells can compensate for loss of ABCA1 more readily than loss of LXRβ to maintain cholesterol homeostasis, cell cycle progression, and cell viability.

### LXRβ activation in dense cells represses mevalonate pathway activity independently of ABCA1

**Figure 5:**
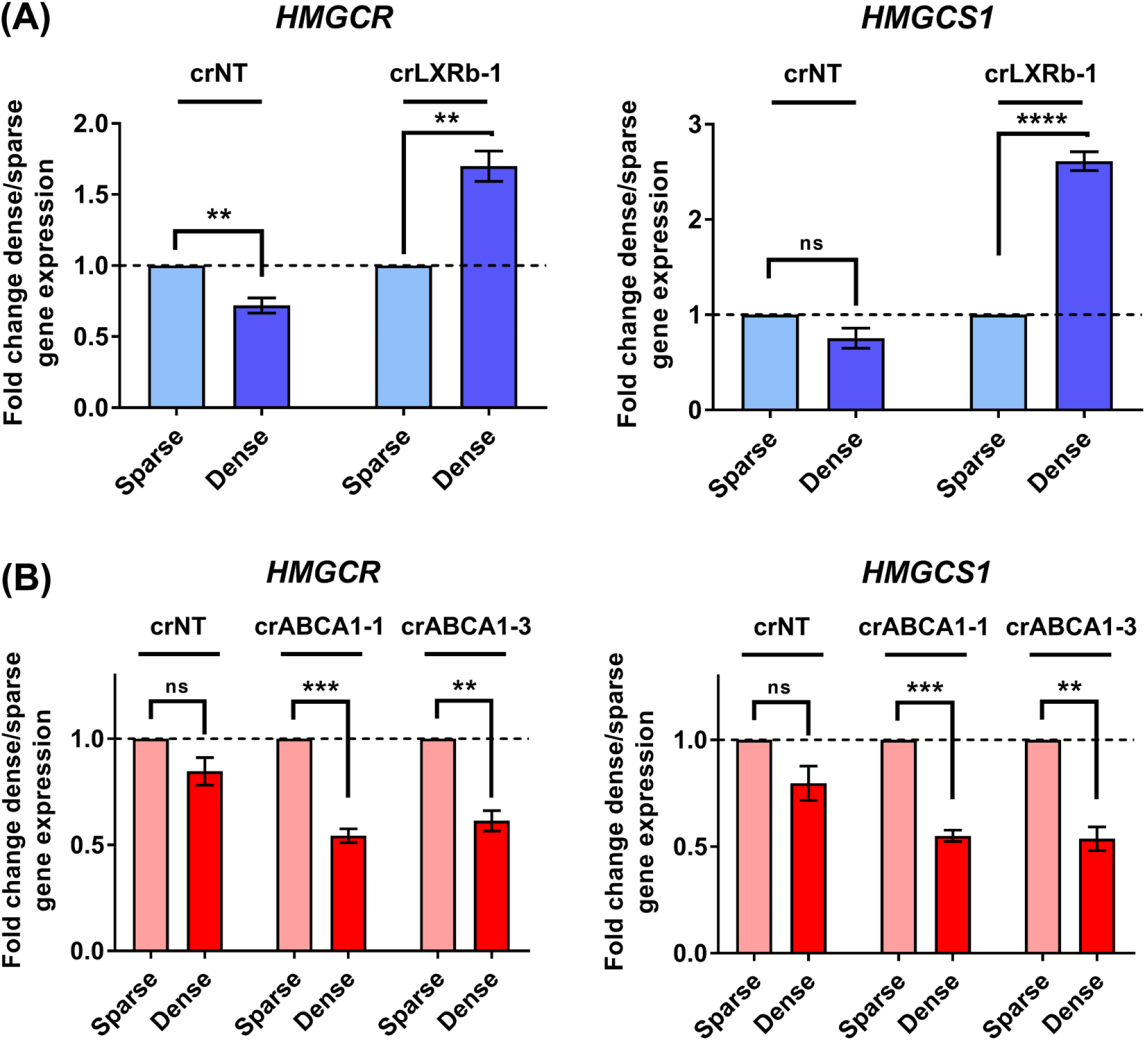
LXRβ and ABCA1 have different effects on feedback regulation of mevalonate pathway. (**A**) Relative gene expression analysis for mevalonate pathway genes *HMGCR* and *HMGCS1* in crNT and crLXRb-1 TS600 glioma cells. Gene expression values were derived from quantitative real time PCR normalized to *GAPDH* and expressed as fold change dense/sparse for crNT and crLXRb-1. Error bars indicates SEM for at least 3 replicates. **p < 0.005, ****p = 0.00008 for multiple t-tests using procedure of Benjamini, Krieger and Yekutieli. (**B**) Relative gene expression analysis for mevalonate pathway genes *HMGCR* and *HMGCS1* in crNT and crABCA1 TS600 glioma cells. Gene expression values were derived as in (A). Error bars indicates SEM for at least 3 replicates. **p < 0.005, ***p < 0.0005 for multiple t-tests using procedure of Benjamini, Krieger and Yekutieli.

Figure 4A shows that cells with decreased ABCA1-mediated cholesterol transport via CRISPR-mediated knockdown of ABCA1 do not accumulate cholesterol at high plating density. In contrast, cells with decreased ABCA1 through CRISPR-mediated knockdown of its transcriptional activator LXRβ amass cholesterol (Fig. 3A). To better understand this seeming paradox, we measured the expression of *HMGCR* and *HMGCS1*, two genes in the mevalonate pathway that are critical for *de novo* cholesterol synthesis and are regulated by cholesterol levels in an SREBP-dependent feedback loop^41^. We observed that while there was little or no cell density-dependent difference in the levels of these genes in cells expressing a non-targeting CRISPR (crNT), cells expressing crLXRb-1 significantly increased levels of these genes at high cell density, while cells expressing crABCA1 decreased them (Fig. 5A,B). This result suggests that the cholesterol accumulation observed in the crLXRβ cells is due to *de novo* synthesis through upregulation of the mevalonate pathway in parallel with less efflux through ABCA1 (compare Figs 3A, 5A). In contrast, the apparent lack of cholesterol accumulation in the crABCA1 cells might be due to a homeostatic balance of accumulation due to decreased efflux simultaneous with a decrease in *de novo* synthesis (compare Figs 4A, 5B). Overall, our data indicate that ABCA1 plays a crucial role in regulating efflux of cholesterol in highly dense glioma cells, but LXRβ signaling maintains safe levels of cholesterol through a more complicated balance of biosynthesis, uptake, and efflux of cholesterol.

### *ABCA1* and *NR1H2* gene expression are not correlated in the TCGA GBM data set

**Figure 6:**
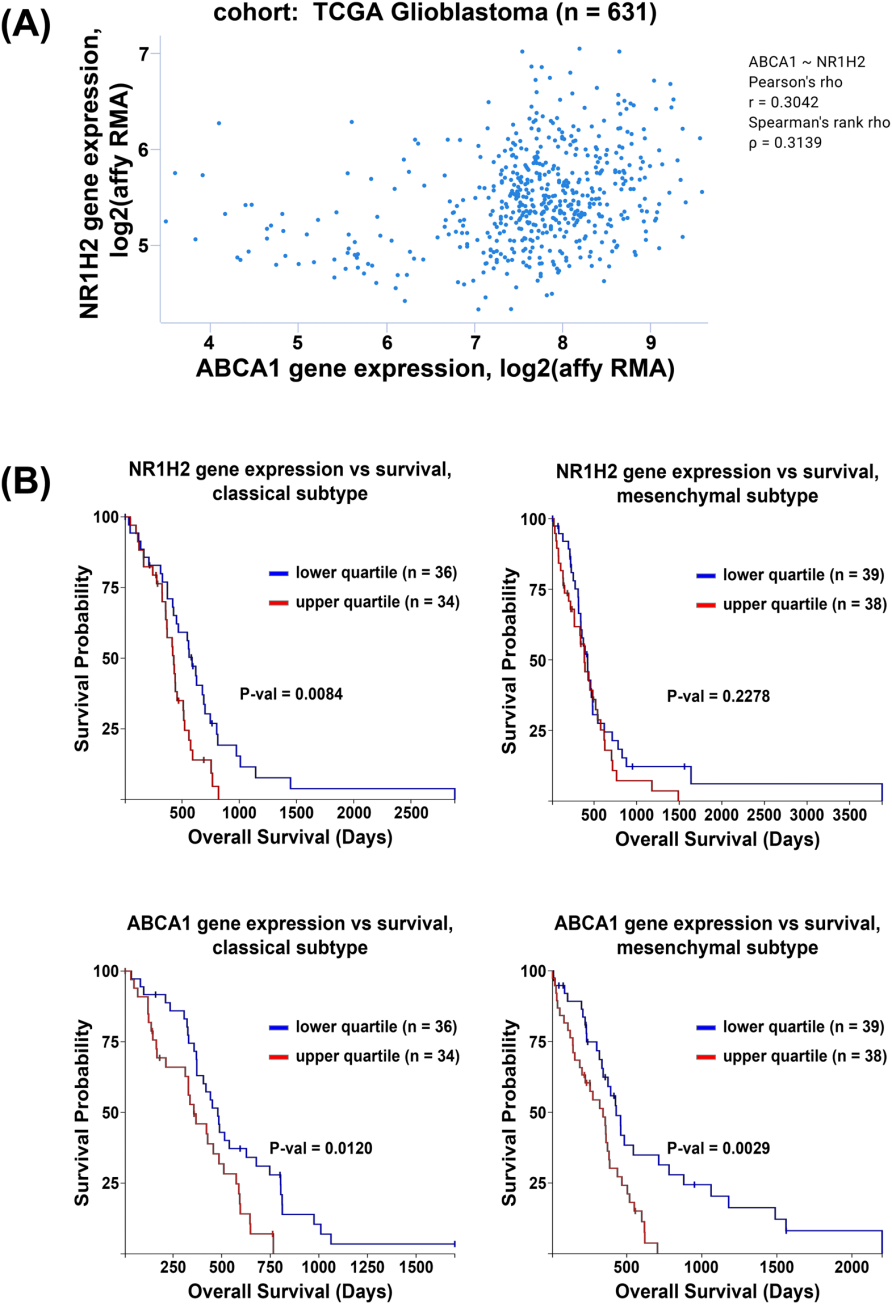
*NR1H2* and *ABCA1* gene expression are not related in GBM patients. (**A**) Pearson correlation of NR1H2 and ABCA1 gene expression in TCGA GBM samples. (**B**) Kaplan-Meier curves for GBM molecular subtypes, classical and mesenchymal, comparing the lowest and highest quartile of *NR1H2* (top row) and *ABCA1* (bottom row) gene expression.

One explanation for the differences in cholesterol homeostasis between the crABCA1 and crLXRβ cells might be due to ABCA1-independent effects of LXRβ in the ABCA1 knock-out cells. To address this, we first looked at *NR1H2* and *ABCA1* gene expression in the TCGA Glioblastoma data set and saw that they are only very weakly correlated (Fig. 6A). We next looked for gene expression correlations with patient survival in the three GBM subtypes, classical, mesenchymal, and proneural^42^. While neither gene related to prognosis in the proneural subtype (data not shown), high expression of either gene correlated with poor prognosis in patients with the classical subtype (Fig. 6B). In contrast, only high expression of *ABCA1* but not *NR1H2* correlated with poor prognosis in the mesenchymal subtype (Fig. 6B). These data showing that these genes correlate with poor prognosis in different subsets of patients suggest that these genes might have important, independent functions.

### LXRβ controls immune phenotypes and glycerophospholipid accumulation in glioma tumor neurosphere cells

**Figure 7:**
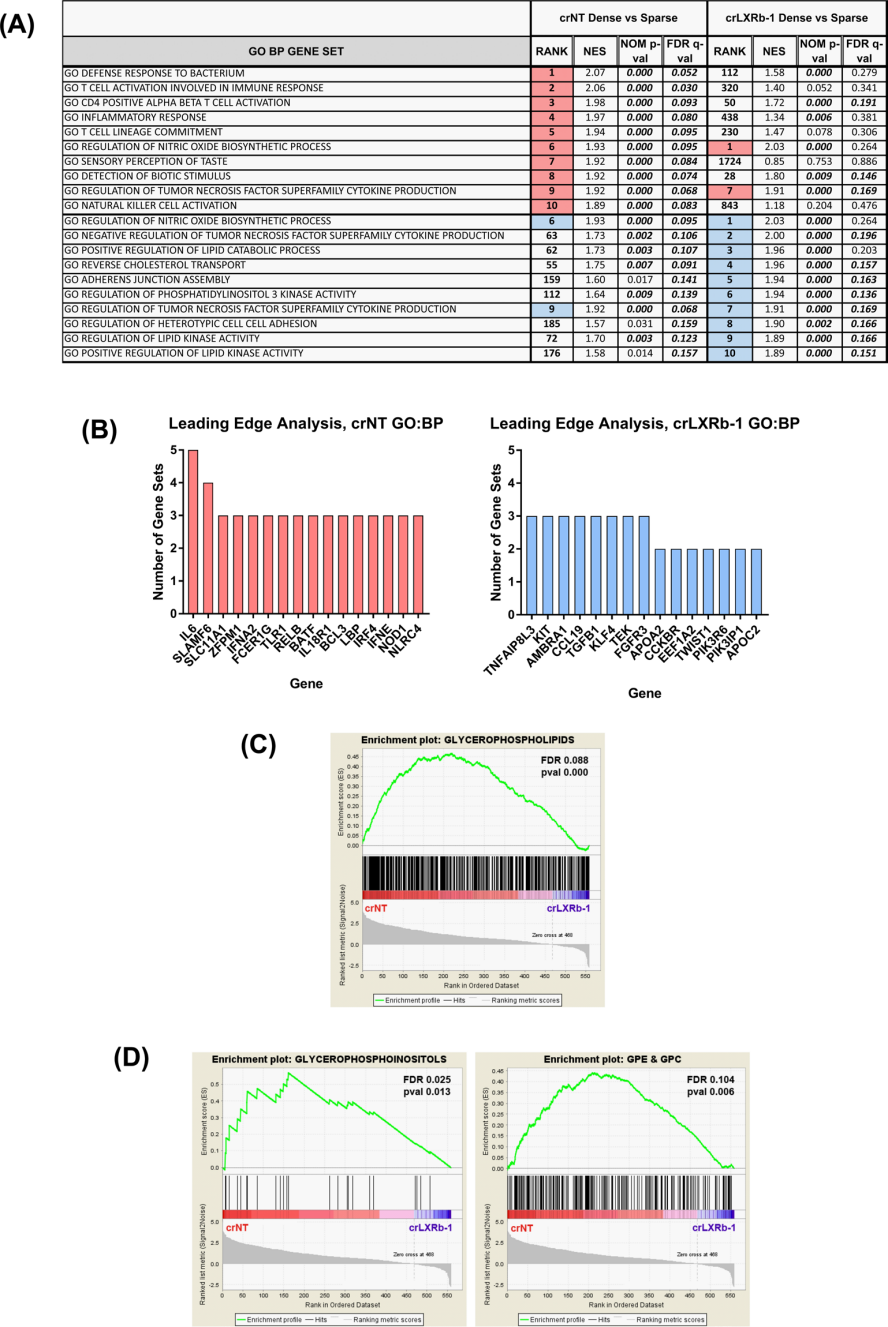
LXRβ regulates immune response and glycerophospholipids accumulation in glioma tumor initiating cells. (**A**) Gene Set Enrichment of Gene Ontology Biological Processes in TS600 crNT dense vs sparse compared to crLXRb-1 dense vs sparse. Pink sets are enriched in crNT cells and blue are enriched in crLXRb-1. (**B**) Leading edge analysis of genes shared between enriched gene sets for crNT (pink bars) and crLXRb-1 (blue bars) in (A). (**C**) crNT and crLXRb-1 cells were subjected to lipidomics profiling, then enrichment analysis was performed using LIPID MAPS categories. The category “Glycerophospholipids” is shown. (**D**) Enrichment analyses of the LIPID MAPS mainclass subsets of “Glycerophospholipids”. The mainclasses “Glycerophosphoinositols” and “Glycerophosphoethanolamines/Glycerophosphocholines (GPE/GPC)” is shown.

To further define ABCA1-independent roles of LXRβ in glioblastoma, we compared gene set enrichment analyses (GSEA) for crLXRb-1 and crNT cells at low and high cell density^43^. Remarkably, 8 of the top 10 enriched GO Biological Process gene sets enriched in dense crNT cells relative to sparse were involved in the immune response; only 2 of these were also in the top 10 for the crLXRb-1 cells (Fig. 7A,B, highlighted in pink). Conversely, top gene sets enriched in the dense crLXRb-1 cells were predominantly involved in lipid kinase activity and cell-cell adhesion; only 2 of these were also in the top 10 for the crNT cells (Fig. 7A,B, highlighted in blue).

Because of the important role of LXRβ in cholesterol homeostasis and lipid regulation, we performed lipidomics on dense crNT and crLXRb-1 cells. We saw that of the LIPID MAPS categories fatty acyls, glycerolipids, glycerophospholipids, sphingolipids, and sterols, only the glycerophospholipids were significantly decreased in the crLXRb-1 cells relative to crNT (Fig. 7C)^44^. Further subdivision of this category revealed that glycerophospoinositols (GPIs) and glycerophosphoethanolamines/glycerophosphocholines (GPE/GPC, which couldn’t be resolved well) were decreased in the LXRβ CRISPR cells, but not glycerophosphoserines, glycerophosphates, or glycerophosphoglycerols (Fig. 7D). These data suggest that LXRβ plays diverse roles in glioma biology, including cholesterol homeostasis, immune responses, and glycerophospholipid balance.

## Discussion

Dysregulated metabolism and therapeutic resistance are important hallmarks of GBM. Exploration of the key metabolic genes *G6PD, TKT*, *FASN* and their regulatory pathways have already been reported^45,46^, however, not much attention has been directed towards cholesterol biology. Cholesterol is exchanged in the form of lipoproteins between cells or is generated *de novo* by the mevalonate pathway^47^. ATP binding cassette transporters such as ABCA1 and ABCG1 mediate lipoprotein efflux, with low-density lipoprotein receptor (LDLR) proteins regulating the subsequent uptake of lipoproteins^48^. Cellular lipoprotein efflux and uptake are tightly regulated by Liver X receptors (LXRα and LXRβ), which heterodimerize with retinoid X receptors (RXRs) to regulate transcription. Cholesterol derivatives (oxysterols and cholestenoic acids) are ligands of LXR:RXR heterodimers, promoting transcriptional activation of genes that direct the transport of lipids. Previously, a study published by our group showed that glioma cells have dysregulated cholesterol synthesis relative to normal astrocytes^15^. We also reported that cholesterol synthesis in glioblastoma tumor initiating cells differ from primary astrocytes in that it is independent of cell cycle control and cell density. Here, our findings indicate that glioma tumor initiating cells depend on LXRβ to maintain cholesterol homeostasis, cell viability, and cell proliferation at high cell density, conditions under which *de novo* cholesterol synthesis is constitutively activated.

**Figure 8:**
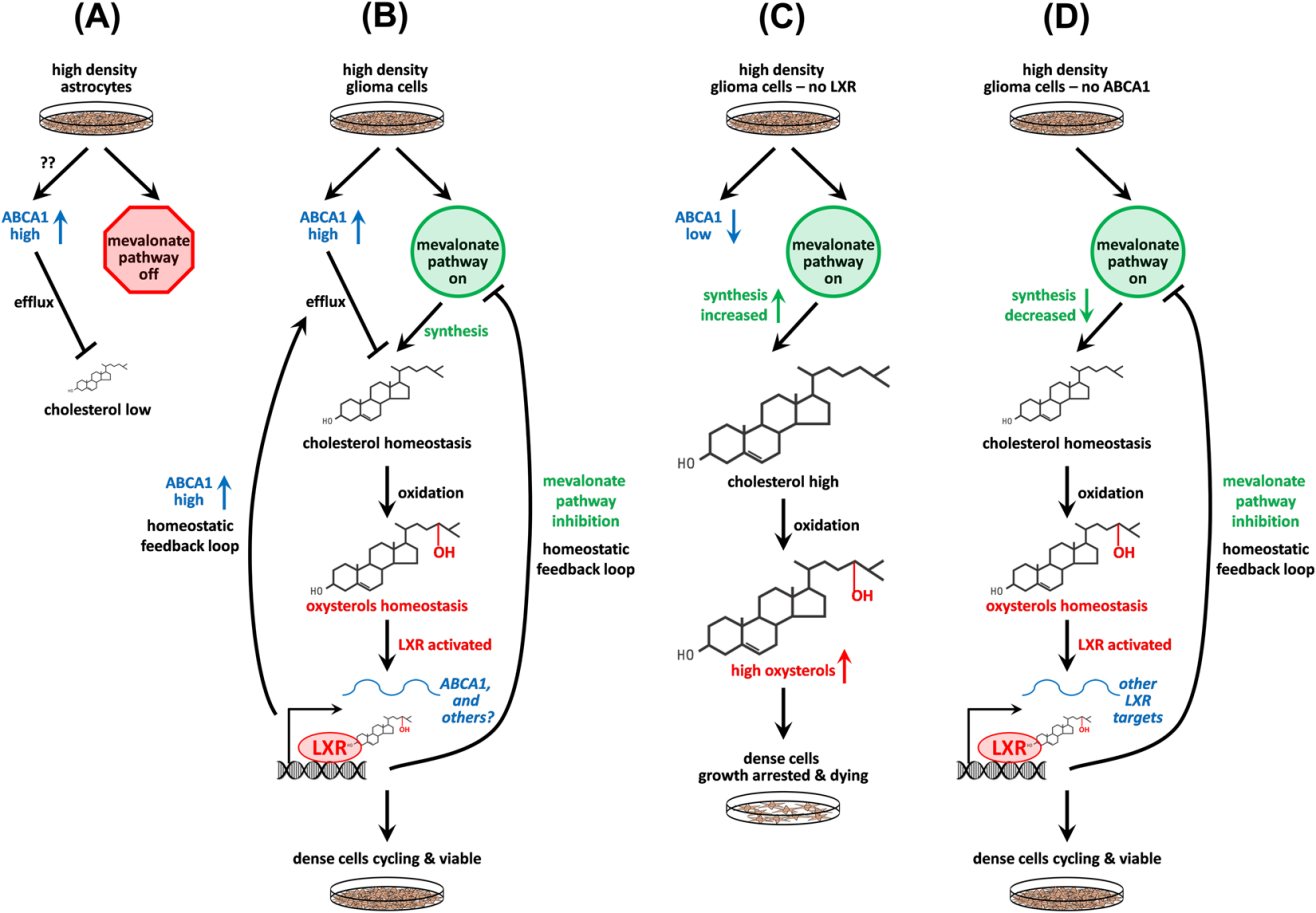
Summary: cholesterol homeostasis pathways in astrocytes and glioma cells. (**A**) At high plating density, normal astrocytes lower cholesterol levels compared to sparsely plated cells by both increasing ABCA1 (Figure S1) and by decreasing *de novo* synthesis through the mevalonate pathway^15^. The factors that activate ABCA1 in dense normal astrocytes are not defined, but might involve inhibition of FAK^49^. (**B**) Glioma tumor initiating cells at high density keep cholesterol levels equivalent to sparse through the net effects of multiple feedback loops. Unlike normal astrocytes, they generate new cholesterol by keeping the mevalonate pathway on^15^. This cholesterol in turn is oxidized to oxysterols (Fig. 2), which act as activating ligands for LXR. Activation of LXR then in turn keeps cholesterol levels in check (Fig. 3) by increasing cholesterol efflux via upregulating ABCA1 RNA and protein (Figs 1 and 2) and through inhibition of the mevalonate pathway (Fig. 5). This enables the cells to survive and proliferate under conditions in which normal astrocytes cells are contact inhibited (Fig. 3 and ref.^15^). (**C**) In the absence of LXR, intracellular cholesterol levels are high (Fig. 3), likely due to low ABCA1 levels (Fig. 2) and increased activity in the mevalonate pathway (Fig. 5). This leads to decreased cell proliferation and viability, possibly due to toxic levels of cholesterol and/or oxysterols (Fig. 3). (**D**) In the absence of ABCA1, net cholesterol levels are equivalent in sparse and dense cells and the glioma cells continue to proliferate and are viable at high density (Fig. 4). While decreased ABCA1 should result in an accumulation of cholesterol due to decreased efflux, cholesterol is maintained at levels compatible with cell viability through feedback inhibition of the mevalonate pathway (Fig. 5), possibly through increased LXR activation by accumulated oxysterols, or though feedback inhibition of transcription factors such as SREBP^41^.

Figure 8 summarizes our findings. At high cell density, normal astrocytes reduce intracellular cholesterol by both upregulating the cholesterol efflux transporter ABCA1, and by reducing expression of genes in the mevalonate pathway^15^ (Fig. 8A). We cannot explain how these cells upregulate ABCA1 as we found no evidence for cholesterol-mediated activation of LXRβ, though recent studies have found high cell density can activate ABCA1 through the inhibition of FAK^49^. In contrast, glioma tumor initiating cells plated at high density maintain cholesterol at levels compatible with cell proliferation and viability by (1) keeping *de novo* synthesis on through the mevalonate pathway and by (2) activating LXRβ to upregulate cholesterol efflux through ABCA1 and pathways involved in lipid metabolism and immune regulation (Fig. 8B). Cells deficient in LXRβ due to CRISPR-mediated knockdown had high intracellular cholesterol levels, presumable due to decreased efflux through ABCA1 and/or mevalonate pathway feedback inhibition (Fig. 8C). These cells were compromised in viability and proliferation, though we have not established whether this is due to direct regulation of these activates by LXR and/or indirect via changes in cholesterol levels. Interestingly, cells with CRISPR-mediated knockdown of ABCA1 and plated at high density maintained intracellular cholesterol at high cell density, possibly through the net effect of decreases in both efflux and de *novo* synthesis through the mevalonate pathway (Fig. 8D). Despite these changes in cholesterol dynamics, the crABCA1 cells were viable and continued to proliferate at high density and thus did not recapitulate the phenotypes we observed with crLXRβ, suggesting that ABCA1 might be a necessary, but not sufficient, effector of these functions. Villa *et al*. published a study where they found that the activation of LXR via LXR-623 is lethal to GBM cells, further emphasizing a role LXRβ plays in maintaining cholesterol homeostasis^35^. A dissection of the relationship between *NR1H2* and *ABCA1* gene expression in the TCGA Glioblastoma data set shows that only very weak correlation, which is in congruence with our experimental findings.

To further refine the role of LXRβ in glioblastoma, we compared gene set enrichment analyses (GSEA) for crLXRb-1 and crNT cells at low and high cell density. Top gene sets enriched in dense cells with intact LXRβ were predominantly involved in the immune response, suggesting that an important role for LXRβ in glioblastoma growth might be immune evasion, which could either be directly or indirectly related to its role in cholesterol homeostasis. Besides the well-known role of cholesterol in the biology of cellular structures such as membranes and lipid rafts, recent work has implicated its role in the immune response. For example, higher anti-tumor activity of CD8 + T cells could be achieved by modulating cholesterol levels^50^ and an analogue of cholesterol can act as a negative regulator for TCR signaling^51^. Our lipidomic data showed that CRISPR-modified LXRβ tumor initiating cells had a significant decrease in glycerophospholipids. Upon further subdivision of the category, we found that specifically glycerophosphoinositols and not glycerophosphocholines/glycerophosphoethanolamines were decreased in the LXRβ CRISPR cells. Studies recently published by Patrussi *et al*. and Reboldi *et al*. showed that glycerophosphoinositols and 25-hydroxycholesterol activate T cells and host immune responses by upregulating IL-1β, which has a well-characterized role in tumor inflammation^52,53^. Together, these findings support our conclusions that LXRβ plays diverse roles in cancer cell biology, including cholesterol homeostasis, immune responses, cell cycle regulation, and therapeutic resistance.

This study provides better understanding of aberrant metabolic programming in GBM based on differential cellular density. From a translational point of view, comprehensive characterization of GBM tumors based on their cellular and molecular properties such as cell density, genetic alterations, cholesterol and lipid accumulation, might redefine therapeutic regimens for GBM. Unlike other organs, the brain primarily derives its cholesterol from *de novo* synthesis rather than the diet^47^, thus therapeutic strategies aimed at disrupting cholesterol homeostasis are likely to exhibit inherent specificity in GBM tumors.

## Methods

### Cells and cell culture

All cells were cultured at 37 °C and 5% CO_2_. Glioma tumor initiating cells derived from surgically resected patient tumors were obtained from Cameron Brennan (MSKCC) and were cultured in DMEM:F12 (HyClone) with 1x B-27 Supplement medium without Vitamin A(Life Technologies), 10 ng/mL EGF (Peprotech), 10 ng/mL bFGF (R&D Systems), Primocin (InvivoGen) and 2 μg/mL heparin (Sigma). Sparse cells were cultured at 50,000 cells/mL and dense cells were cultured at 300,000 cells/mL and grown as spheroids in uncoated cell culture flasks. Tumor initiating cells were only cultured for a maximum of 2 months. Normal human astrocytes (NHA) were purchased from ScienCell Research Laboratories, cultured in Astrocyte Medium (ScienCell Research Laboratories), and passaged by trypsinization with TrypLE Express (Life Technologies). NHAs were used until passage 12.

### Microarrays

Cells were grown at sparse and dense conditions for 72 hours prior to harvesting with the NucleoSpin RNA Plus Kit (Clontech). All RNAs had RIN values greater than 8.2 as assessed on an Agilent BioAnalyzer. RNA was 3′ labeled with a Genechip 3′ IVT Express Kit (Affymetrix) and hybridized to Affymetrix PrimeView Human Gene Expression Arrays. Microarray data were analyzed using Partek Genomics Suite as follows: arrays were normalized using RMA quantile normalization with median polish probeset summarization. Only arrays with Relative Log Expression Ratios of less than 0.61 were used in the analysis. RNA preparation dates were determined by Welch’s ANOVA to contribute to batch effects and were removed in Partek Genomics Suite. Fold change of gene expression was measured with 1-way ANOVA. Probesets were collapsed to Gene ID’s by lowest p-value. Microarray data are available at GEO (https://www.ncbi.nlm.nih.gov/gds), Accession GSE79097. Heat maps were generated using CIMminer (https://discover.nci.nih.gov/cimminer/)^54^.

### Quantitative real-time PCR

Glioma tumor initiating cells were grown at sparse and dense conditions for 72 hours in suspension prior to harvesting with the NucleoSpin RNA Plus Kit (Clontech). cDNA was generated using the iScript Advance cDNA synthesis kit for RT (Bio-Rad), and PCR was performed using the KAPA SYBR FAST qPCR kits (KAPA Biosystems) on an Applied Biosystems 7500 Real Time PCR system. Fold-change gene expression for dense relative to sparse was calculated by normalizing to GAPDH followed by the Comparative C_T_ method. Statistical analyses and graphing was performed in GraphPad Prism 7.01. All primers (Sigma KiCqStart) were validated to have an efficiency between 90 and 110%. Primers sequences:

ABCA1: Sense - AACAAGCCATGTTCCCTCAG; Antisense – GACGCAAACACAAAAGTGGA

NR1H2: Sense – ATCCACTATCGAGATCATGC; Antisense - GTCCTTCAAGAAGGTGATAC

NR1H3: Sense – CATGACCGACTGATGTTC; Antisense - CAAACACTTGCTCTGAGTG

HMGCR: Sense – ACTTCGTGTTCATGACTTTC; Antisense - GACATAATCATCTTGACCCTC

HMGCS1: Sense – TTGGCTTCATGATCTTTCAC; Antisense - AATTTAACATCCCCAAAGGC

GAPDH: Sense – ACAGTTGCCATGTAGACC; Antisense – TTTTTGGTTGAGCACAGG.

### Generation of CRISPR knockout glioma tumor initiating cells

See Supplemental Methods for CRISPR design and construction and lentivirus production.

### Cholesterol extraction and quantification

Cholesterol was extracted using a modified Bligh and Dyer method carried out entirely with glass labware. Briefly, TS Glioma cells were plated in suspension and kept for 72 hours. After 72 hours, cells were washed with PBS and cells were counted using the Trypan Blue exclusion assay, 1 × 10^6^ live cells were resuspended in 200 μL PBS and either frozen on dry or immediately processed. To cell suspension, 700 μL of a 2:1 ratio of methanol: chloroform solution was added and the tube vortexed followed by addition of 300 μL of chloroform and a second round of vigorous vortexing. Finally, 250 μL of 1 M NaCl was added and mixed by vortexing. Samples were centrifuged at 3000 × g for 15 minutes at 4 °C and the organic (bottom) phase collected with a Pasteur pipette into a fresh glass test tube. Samples were vacuum dried for 1 to 1.5 hours. Cholesterol was resuspended in 80 μL of 5X Amplex buffer with periodic vortexing for 1 hour, followed by dilution with additional 320 μL water to make 1X buffer concentration. Amplex Red Cholesterol Assay (Life Technologies) was carried out according to manufacturer instructions. Statistical analyses and graphing was performed in GraphPad Prism 7.01.

### 24-Hydroxycholesterol extraction and quantification

Glioma tumor initiating cells were plated in suspension and kept for 72 hours, then washed with PBS and counted using the Trypan Blue exclusion assay, 1 × 10^6^ live cells were resuspended in 1 ml of 95% Ethanol. Extract was then centrifuged at 7000 × g at room temperature for 5 minutes. Supernatant was collected and retained. 1 ml of Ethanol:Dichloromethane (1:1; v/v) was added to the pellet and sonicated for 10 minutes. Extract was centrifuged as described earlier and supernatant was collected and mixed with what we collected earlier. The pooled supernatant sample was then evaporated to dryness with a rotary evaporator. Samples were rehydrated at room temperature by adding 16 μl of 95% ethanol followed by 484 μl of Assay Buffer, this was required to fully solubilize the 24(S)-Hydroxycholesterol present following this sample preparation procedure. 24(S)- Hydroxycholesterol ELISA assay (Enzo Life Sciences) was carried out according to manufacturer instructions.

### SDS-PAGE and Western Blot analysis

Glioma tumor initiating cells were plated in suspension and kept for 72 hours. After 72 hours, cells were washed with PBS, proteins were extracted using RIPA buffer containing Protease and Phosphatase inhibitor cocktails followed by sonication. Protein concentration was measured using colorimetric assay by DC Protein assay (Bio-Rad). 60 μg of protein was loaded into the Novex NuPAGE 3–8% Tris-Acetate Protein gels (Invitrogen). Samples were boiled at 70 °C for 10 mins with loading buffer, except for ABCA1 detection. Proteins were transferred using the Trans-Blot Turbo Transfer System (Bio-Rad). ABCA1 antibody was used at dilution 1:500 from Abcam (Ab18180), β-actin was used at 1:8000 dilution from Sigma (A2066), Vinculin was used at 1:1000 dilution from Cell Signaling (13901). Western blots were developed using Odyssey CLx imaging system from LI-COR. For the membrane fractionation, Mem-PER Plus Membrane Protein Extraction kit (ThermoFisher Scientific) was used as per the manufacturer recommendation.

### BrdU-dilution assay

Glioma cells were plated at sparse and dense confluency and 10 μM BrdU (BD Pharmingen) was added to the cell culture medium for 4 hours. After 4 hours, cells were washed with PBS for once, 1 × 10^6^ cells were collected and stored, and rest of the cells were re plated in the cell culture medium for 72 hours. After 72 hours, cells were collected, counted using the Trypan Blue Exclusion assay and 1 × 10^6^ cells were taken. The cells were washed thoroughly, 700 μL of ice-cold DPBS was added and kept on ice. To fix the cells, 100 μL of ice-cold ethanol was added and vortexed. This step was repeated two more times. Cells were kept in ethanol for 3 hours at 4 °C. After that, cells were centrifuged, and supernatant was removed. 1 mL of 2N HCl/Triton X-100 was added and incubated at room temperature for 30 minutes. Cells were centrifuged and supernatant was removed and resuspended in 1 ml of 0.1 M Na_2_B_4_O_7_, pH 8.5 to neutralize the cells and kept for 10 mins. Cells were incubated with Alexa flour 647- Anti-BrdU (Biolegend) as recommended and treated with RNase A overnight. Cells were then washed with DPBS and resuspended in PI solution for whole cell staining for 20 minutes. Cells were filtered using a cell strainer and flow cytometry was performed using BD LSRFORTESSA (BD Biosciences). Statistical analyses and graphing were performed in GraphPad Prism 7.01.

### CellTox Green Cytotoxicity assay

TS glioma cells were plated at different cell number in a black 96-well plate and kept for 72 hours. After 72 hours, assay was performed as per the manufacturer’s protocol. (Promega)

### Lipidomics

7-HOCA was measured by Metabolon as described in Kambach *et al*.^15^. Complex lipid metabolomic analysis was performed by the NIH West Coast Metabolomics Center by CSH-ESI QTOF MS/MS (metabolomics.ucdavis.edu). Metabolites were identified using The Metabolomics Workbench (www.metabolomicsworkbench.org) searching RefMet with a mass tolerance of +/− 0.05 m/z. Lipid categories were assigned using Lipid Maps (www.lipidmaps.org)^44^. Lipid enrichment analysis was performed by creating “genesets” using Lipid Maps categories in GSEA^43^.

### Patient data analysis

Data from the TCGA Glioblastoma data set was downloaded from the UCSC Xena Functional Genomics Browser (https://xena.ucsc.edu). *NR1H2* and *ABCA1* gene correlations were visualized and quantitated with the TCGA Glioblastoma AffyU133A data using Pandas (https://pandas.pydata.org) in Jupyter Lab. Kaplan-Meier curves for each gene expression subtype were generated using the UCSC Xena Browser with the TCGA Glioblastoma AffyU133A data set, by quartile.

## Supplementary information

Supplementary Materials

## Data Availability

The datasets generated during and/or analysed during the current study are available from the corresponding author on reasonable request.
