# Supplementary material for: LXRβ controls glioblastoma cell growth, lipid balance, and immune modulation independently of ABCA1
Source: Sci Rep. 2019 Oct 29;9:15458. doi: 10.1038/s41598-019-51865-8 (PMC6820787; doi:10.1038/s41598-019-51865-8)
Supplement: Supplementary file 1 — Supplementary Materials [file 41598_2019_51865_MOESM1_ESM.pdf]

SUPPLEMENTAL MATERIALS for:

LXRβ controls glioblastoma cell growth, lipid balance, and immune modulation independently of ABCA1

Deven Patel, Fahim Ahmad, Diane M. Kambach, Qian Sun, Alan S. Halim, Tamalee Kramp, Kevin A. Camphausen, Jayne M. Stommel*

Radiation Oncology Branch, National Cancer Institute, National Institutes of Health
Bethesda, MD 20892 USA

* correspondence to jayne.stommel@nih.gov

Supplemental Methods

Patient data analysis: Data from the GTEX data sets were downloaded from the UCSC Xena Functional Genomics Browser (https://xena.ucsc.edu). GTEX data for *NR1H2* and *NR1H3* in tissues that had at least 100 samples were plotted using Matplotlib (https://matplotlib.org) and Seaborn (https://seaborn.pydata.org) in Jupyter Lab (https://jupyterlab.readthedocs.io/en/stable/).

CRISPR design and construction: CRISPR sequences in NR1H2 were designed using the Broad Institute sgRNA designer at https://portals.broadinstitute.org/gpp/public/analysis-tools/sgrna-design, then compared with crispr.mit.edu. The following sequences were tested:

CRISPR #	sgRNA sequence	PAM Sequence	Broad sgRNA Score	MIT Guide Specificity Score	exon	best in our assays
LXRb-1	GCTGCTGCAACGCCTCCACG	CGG	0.9462	72	9	x
LXRb-2	CGAGGGTGTCCAGCTAACAG	CGG	0.7826	79	6	x
LXRb-3	GCCTGCCTCAGATCCACCAG	GGG	0.6309	58	6	
LXRb-4	AATGATCCAGCAGTTGGTGG	CGG	0.6175	68	6	
LXRb-5	GGACCCTCCTCCTTTACAGT	GGG	0.6102	99	4	

CRISPR sequences in ABCA1 were designed using the CHOPCHOP sgRNA designer at http://chopchop.cbu.uib.no. The following sequences were tested:

CRISPR #	sgRNA sequence	PAM sequence	CHOP CHOP rank	MIT Guide Specificity Score	exon	best in our assays
ABCA1-1	GAACACTTCCTTGGGTTCAG	GGG	2	65	4	x
ABCA1-3	GCCTGTTCTCAGATGCTCGG	AGG	4	83	5	x
ABCA1-4	GCTGTGTCTCGTATTGTCTG	CGG	5	79	9	
ABCA1-5	GTCTGCGGGCATCCCGAGGG	AGG	6	78	9	

Oligonucleotides of the sgRNA sequences were cloned into lentiCRISPRv2 (Feng Zhang, Addgene plasmid # 52961; http://n2t.net/addgene:52961; RRID:Addgene_52961) or a modified

lentiCRISPRv2 with GFP replacing the puromycin resistance gene (deposited in Addgene), using the Zhang lab lentiCRISPRv2 and lentiGuide oligo cloning protocol. Maps were generated in SnapGene.

Lentivirus production: Briefly for all the CRISPR constructs, $1X10^7$ HEK 293-T cells were cultured in a 10 cm2 plate, which was coated with collagen, in DMEM medium supplemented with 10% FBS one day prior to the transfections. The next day, cells were transferred into 15 mL OptiMEM. Transfection mixture was made containing 49.2 µL of Lipofectamine ® 2000 (Thermo), Lenti Vector (12 µg), psPAX2 (9 µg), pMD2.VSVG (3.60 µg) and 2.95 mL of OptiMEM was added to make 3 mL of total volume. After 20 mins, the transfection mixture was added to the cells and incubated overnight. The next day, cell culture medium was changed to DMEM with 10% FBS. Medium was collected at 24 and 32 hours after the medium change, pooled, and filtered over a 0.45 micron filter. The virus was concentrated using ultracentrifugation in SW41 T1 rotor at 23,000 rpm for 2.5 hours, and the pellet was resuspended in glioma stem cell medium. Because lentivirus can be toxic to glioma tumor initiating cells, a functional virus titer was performed by infecting cells with a dilution series of virus in 8 ug/mL polybrene, then performing a CellTiter-Glo (Promega) viability assay to determine the maximal concentration of virus that can be given to cells without effecting cell viability. Transduced glioma cells were either selected in puromycin or Fluorescence Activated Cell Sorting (FACS) sorted for GFP expression after 3-4 days. Cells were only cultured for 2 months maximum.

Supplemental Figure Legends

Figure S1. ABCA1 levels in sparse and dense normal human astrocytes. (A) Relative gene expression analysis for *ABCA1* in normal human astrocytes (NHA). Gene expression values were derived from quantitative real time PCR normalized to GAPDH and expressed relative to the 24 hour time point for sparse cells. Error bars indicates SEM for at least 3 replicates. * = $p < 0.05$, ** = $p < 0.005$, *** = $p < 0.0005$ versus 24 hour sparse paired t-test. **(B)** Western blot analysis of ABCA1 and β-actin in NHAs comparing sparse vs. dense conditions. This image is representative of 3 biological replicates. **(C)** Densitometry of ABCA1 protein in (B).

Figure S2. *NR1H2* and *ABCA1* CRISPR design and validation. (A) Expression levels of *NR1H2* and *NR1H3* in normal tissues in the Genotype-Tissue Expression (GTEx) data set. **(B)**

Map of *NR1H2* showing locations of tested CRISPR RNAs. CRISPR sequences used in this manuscript are highlighted with an asterisk. **(C)** PCR of LXRβ target genes in LN340 cells treated with 5 μM GW3965 for 24 hrs and expressing each of 5 tested LXRβ CRISPR constructs. Values are fold change of GW3965 treated relative to crNT untreated, normalized to GAPDH. **(D)** Map of ABCA1 showing location of tested CRISPR RNAs. CRISPR sequences used in this manuscript are highlighted with an asterisk. **(E)** Western blot analysis of ABCA1 and vinculin in TS543 crNT and crABCA1-1,3,4, and 5 glioma cells treated with 5 μM GW3965 or DMSO for 24 hrs. Data shown are representative of at least 2 biological replicates.

(A)

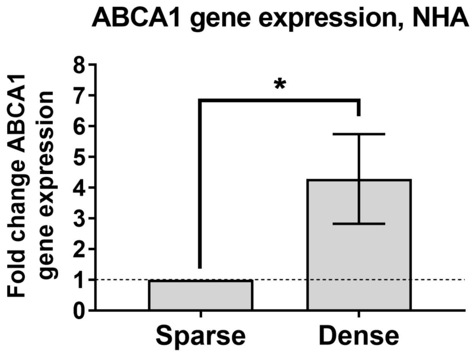
ABCA1 gene expression, NHA
Fold change ABCA1 gene expression
8
7
6
5
4
3
2
1
0
*
Sparse
Dense

(B)

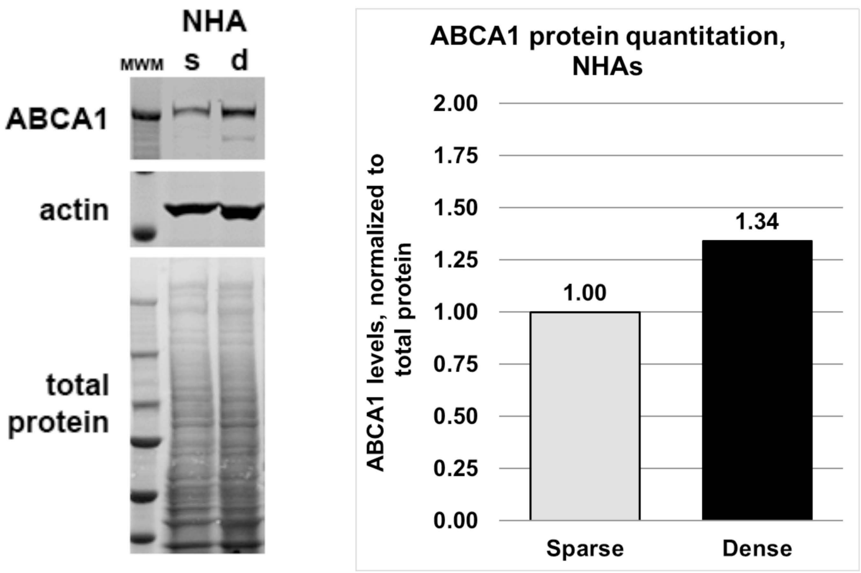
NHA
MWM
s
d
ABCA1
actin
total protein
ABCA1 protein quantitation, NHAs
ABCA1 levels, normalized to total protein
2.00
1.75
1.50
1.25
1.00
0.75
0.50
0.25
0.00
1.00
1.34
Sparse
Dense

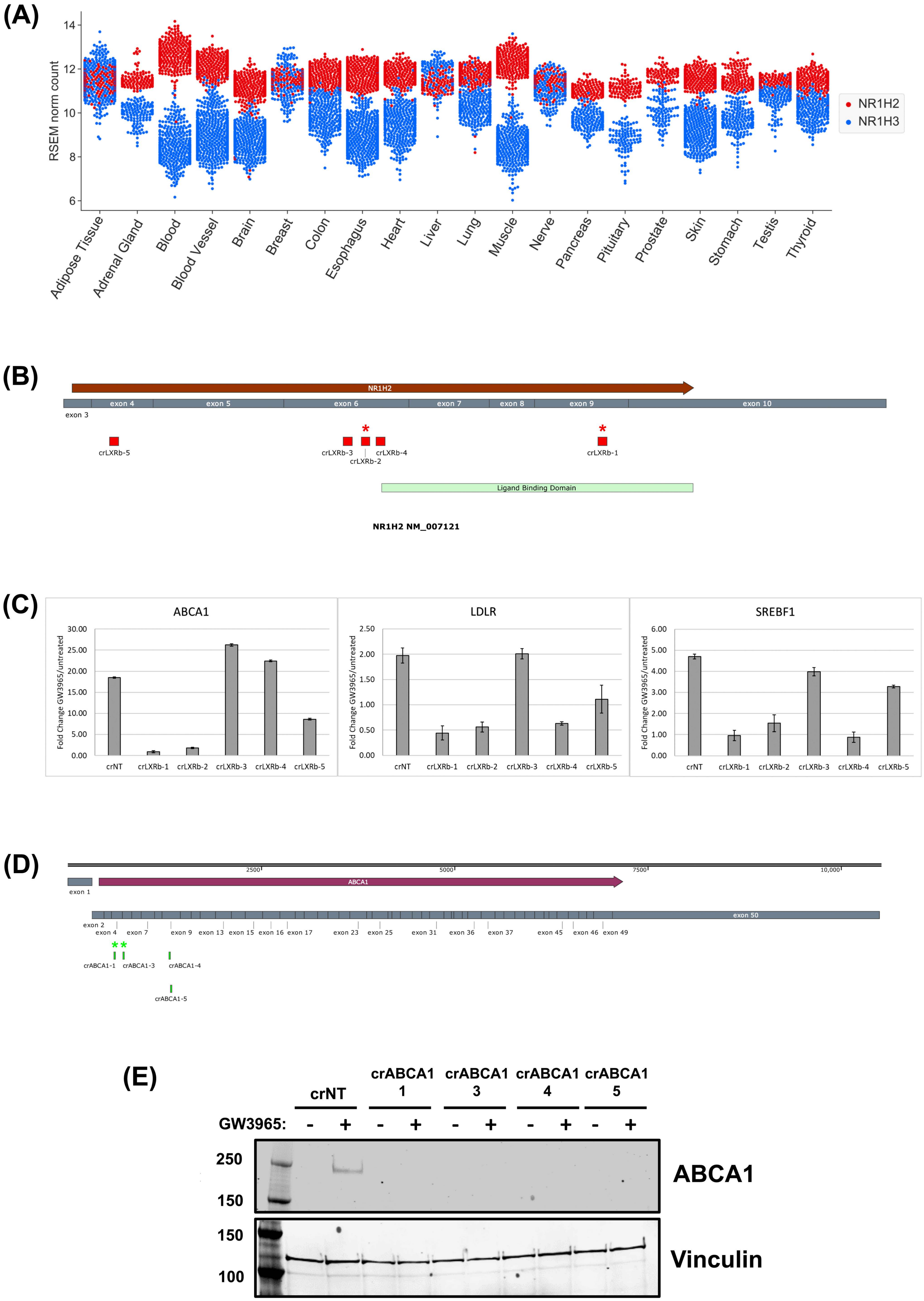
(A)
RSEM norm count
14
12
10
8
6
NR1H2
NR1H3
Adipose Tissue
Adrenal Gland
Blood
Blood Vessel
Brain
Breast
Colon
Esophagus
Heart
Liver
Lung
Muscle
Nerve
Pancreas
Pituitary
Prostate
Skin
Stomach
Testis
Thyroid
(B)
NR1H2
exon 3
exon 4
exon 5
exon 6
exon 7
exon 8
exon 9
exon 10
crLXRb-5
crLXRb-3
crLXRb-2
crLXRb-4
crLXRb-1
Ligand Binding Domain
NR1H2 NM_007121
(C)
ABCA1
LDLR
SREBF1
Fold Change GW3965/untreated
30.00
25.00
20.00
15.00
10.00
5.00
0.00
2.50
2.00
1.50
1.00
0.50
0.00
6.00
5.00
4.00
3.00
2.00
1.00
0.00
crNT
crLXRb-1
crLXRb-2
crLXRb-3
crLXRb-4
crLXRb-5
(D)
2500
5000
7500
10,000
exon 1
ABCA1
exon 50
exon 2
exon 4
exon 7
exon 9
exon 13
exon 15
exon 16
exon 17
exon 23
exon 25
exon 31
exon 36
exon 37
exon 45
exon 46
exon 49
crABCA1-1
crABCA1-3
crABCA1-4
crABCA1-5
(E)
crNT
crABCA1 1
crABCA1 3
crABCA1 4
crABCA1 5
GW3965: - + - + - + - + - +
250
150
ABCA1
150
100
Vinculin
